# Methylphenidate and Long‐Term Risk of Cardiovascular Diseases: A Nationwide Cohort Study

**DOI:** 10.1002/pds.70493

**Published:** 2026-10-11

**Authors:** Christelle Berthod, Thomas Loppinet, Marietou Ndiaye, Inesse Boussaha, Matthias Jacquet‐Lagreze, Behrouz Kassaï‐Koupai

**Affiliations:** ^1^ Clinical Investigation Center CIC‐1407, UMR 5558, Inserm Hospices Civils de Lyon Bron France; ^2^ Pediatric Emergency Unit Hospices Civils de Lyon Bron France; ^3^ Department of General Pediatrics Hospices Civils de Lyon Bron France; ^4^ Department of Anesthesia and Intensive Care, Louis Pradel Cardiology Hospital Hospices Civils de Lyon Bron France; ^5^ Lyon Est Faculty of Medicine Claude Bernard University Lyon 1 Lyon France; ^6^ CarMeN Laboratoire, Inserm UMR 1060 Université Claude Bernard Lyon 1 Lyon France; ^7^ Department of Pharmacotoxicology Hospices Civils de Lyon Lyon France

**Keywords:** attention deficit hyperactivity disease, cardiovascular diseases, cardiovascular risk, children, long‐term effect, methylphenidate, real‐world data

## Abstract

**Purpose:**

Cardiovascular safety of methylphenidate remains a matter of debate.

**Methods:**

To evaluate the long‐term cardiovascular risk associated with methylphenidate use, a cohort study was conducted using the French National Health Data System. New users were defined by a first methylphenidate dispensation (index date) between January 1, 2014, and December 31, 2023. Each new user was matched with three nonusers by sex and by month and year of birth. Individuals with prior cardiovascular disease or relevant cardiovascular comorbidities were excluded. The primary outcome was cardiovascular disease, defined by International Classification of Diseases codes or antihypertensive drug use. Cox proportional hazards models estimated adjusted hazard ratios (HRs), accounting for demographic and clinical covariates. Negative control outcomes were used to assess residual confounding, allowing the derivation of calibrated HRs and confidence intervals.

**Results:**

A total of 212 216 new users and 636 648 matching nonusers were included. Mean age was 13.7 years and 72% were males. Over a follow‐up of ~4 years, 3858 cardiovascular diseases occurred (0.45%): 1960 in new users (9.2 per 1000) and 1898 in nonusers (3 per 1000). The adjusted HR was 2.37 (95% CI [2.21; 2.54]), with a calibrated HR of 1.85 (95% CI [1.55; 2.21]).

**Conclusions:**

The results suggest that methylphenidate use may be associated with an increased risk of cardiovascular diseases. Although the calibrated estimates indicate attenuation due to residual confounding, the association remained statistically significant. This supports recommendations for cardiovascular monitoring during treatment.

## Keypoints

- Concerns about an increased risk of heart problems with methylphenidate come from its chemical structure, which is related to amphetamines, and from evidence that it can raise blood pressure and heart rate.
- Previous studies have reported mixed results, so the question of cardiovascular risk is still open
- Our study, based on a large real‐world administrative database, found that methylphenidate new users had about a 2.37 times higher risk of developing cardiovascular conditions compared to those not exposed.
- Because retrospective cohort studies using large databases can have unmeasured biases, we assessed residual bias, which lowers the estimated risk increase to around 1.8.
- Follow‐up ranged from a few days to 10 years, with an average of 4 +/− 2.5 years. It would be useful to repeat the study in 10 years to better assess long‐term risk.

## Plain Language Summary

Our objective was to determine whether exposure to methylphenidate is associated with a long‐term cardiovascular risk. Although randomized clinical trials have not identified such a risk in the short term, it remains important to assess whether this treatment remains safe when used over longer periods in real‐world settings.

To address this question, we studied all individuals younger than 43 years who were exposed to methylphenidate in France between 2014 and 2023, using a large national healthcare reimbursement database. Each treated individual was matched with three similar individuals who were not exposed to the drug.

We found that individuals exposed to methylphenidate had approximately a 2.4‐fold higher risk of developing cardiovascular disease compared with nonexposed individuals. Follow‐up duration ranged from a few days to nearly 10 years, with an average follow‐up of about 4 years.

However, observational cohort studies may be affected by bias. We therefore applied advanced methods to better control for these biases. After accounting for residual bias, the true risk is likely closer to a 1.9‐fold increase rather than a 2.4‐fold.

**Abbreviations**
CI: confidence interval
CIP13: the 13‐digit drug identifier code (*Code Identifiant de Présentation*)
HR: hazard ratio
ICD‐10: International Statistical Classification of Diseases 10th Revision
MPH: methylphenidate
SNDS: National Health Data System (Système National de Données en Santé)

## 1 Introduction

Methylphenidate is indicated for children aged 6 years and older with attention‐deficit/hyperactivity disorder (ADHD) when nonpharmacological interventions alone have proven insufficient. Methylphenidate has been reported to increase heart rate and blood pressure, which is pharmacologically plausible given its amphetamine‐like structure [1, 2, 3, 4, 5]. However, it remains unclear whether this increase is modest and clinically insignificant in terms of cardiovascular risk [1, 2, 6, 7], or whether it warrants stricter monitoring and more restricted indications [3, 8, 9]. Although its cardiovascular safety remains a matter of debate. While several studies suggest an increased cardiovascular risk [3, 4, 9, 10, 11, 12, 13, 14], others fail to demonstrate such an association [14, 15, 16]; notably, a Cochrane meta‐analyses concluded that the available evidence was limited and insufficient to draw firm conclusions [9]. Two cohort studies conducted using administrative databases reported an increased long‐term cardiovascular risk [11, 12], but neither formally evaluated the potential impact of residual confounding; this may explain the observed associations [11, 12]. Further research is needed to confirm or not these findings using more robust methodological approaches, including formal assessments of residual bias [17, 18, 19, 20, 21]. We therefore conducted a study using the French National Health Data System (*Système National des Données de Santé*, SNDS) to compare long‐term cardiovascular risk among new users and nonusers of methylphenidate, with an assessment of residual bias.

## 2 Methods

### 2.1 Study Design and Setting

We conducted a nationwide population‐based cohort study using the SNDS to evaluate the long‐term effect of methylphenidate on cardiovascular diseases by comparing matched new users to nonusers in France between 2014 and 2023. Each exposed individual (new methylphenidate user) was matched to three nonusers, based on month and year of birth and sex, and applied negative control outcomes to assess potential residual confounding [18, 19, 21]. The study protocol was approved by the INSERM (*Institut National de la Santé et de la Recherche Médicale Française*). Subjects' privacy and confidentiality were assured. In accordance with French law on personal data protection (*Article R. 1461‐12 du Code de la santé publique*), informed consent was not required, as the data were anonymized.

### 2.2 Data Sources

The SNDS comprises healthcare data for 99% of the population of France (over 67 million individuals) [22]. The SNDS integrates the national inter‐scheme health insurance information system and hospital discharge data, linked through a pseudonymized unique national identifier. The health insurance information system gathers pseudonymized demographic data (including sex, month, and year of birth and death) and claims information, such as dates, dosage, and quantities of outpatient drugs dispensed. Hospital discharge data provide information on public and private hospitalizations with International Statistical Classification of Diseases 10th Revision (ICD‐10) codes for primary and secondary diagnoses, dates of admission and discharge [23]. The source database captures only diagnoses recorded during inpatient hospitalizations; outpatient diagnoses are not available in the underlying database.

### 2.3 Participants

Study population selection was initially based on the following eligibility criteria: affiliation to the general scheme of the French national health insurance system and birth between January 1, 1980, and December 31, 2023. Because medication reimbursement records were progressively implemented across health insurance schemes, data reliability was limited during the early years of the database. Methylphenidate‐exposed individuals were identified by at least one reimbursed dispensation of methylphenidate between January 1, 2009, and December 31, 2023, using the 13‐digit drug identifier code (*Code Identifiant de Présentation*, CIP13), which allows identification of both the drug and packaging characteristics (Table S1). Data were not available before January 1, 2009. Patients were considered as new users of methylphenidate if they had not received a reimbursement for a dispensation between January 1, 2009, and December 31, 2014, reflecting the progressive implementation of reimbursement recording data for methylphenidate during this period. Consequently, some patients whose first recorded reimbursement in the database occurred in 2012 may have received earlier dispensations, particularly in 2011, that were not captured in the database. To minimize exposure misclassification related to this progressive implementation, we excluded all patients with at least one methylphenidate dispensation between 2009 and 2014. New users were eligible for inclusion if they had at least two dispensations reimbursed between January 1, 2014, and December 31, 2023; the index date was defined as that of first dispensation. All individuals without a reimbursed methylphenidate dispensation were considered unexposed. Due to the large number of unexposed individuals, and associated technical constraints, five matched unexposed controls (eligible controls) per exposed individual (new users) were initially identified. Five control individuals were randomly selected from the pool of eligible individuals and matched to those treated by methylphenidate on sex, month, and year of birth. Each nonuser individual can be matched to only one exposed individual. Among the five eligible unexposed individuals, those with any of the following comorbidities were excluded: diagnosis of cardiovascular diseases (using ICD‐10 codes) or an exposure to antihypertensive drugs (using CIP13) before the index date, defined exactly like the primary outcome, or pathologies with known cardiovascular risk (using ICD‐10 codes): sleep apnea, congenital malformations of the circulatory system, Down syndrome, fragile X, glomerular diseases, Kawasaki disease, pediatric inflammatory multisystem syndrome (Table S1). Unexposed controls who died before the index date were also excluded. Unexposed subjects were assigned the index date of their matched exposed subjects. Following this step, three matched unexposed controls without comorbidities (nonusers) were randomly selected for each new user (Figure 1). Follow‐up extended from the index date until the occurrence of the outcome, loss to follow‐up, death, or December 31, 2023, whichever came first.

**FIGURE 1:**
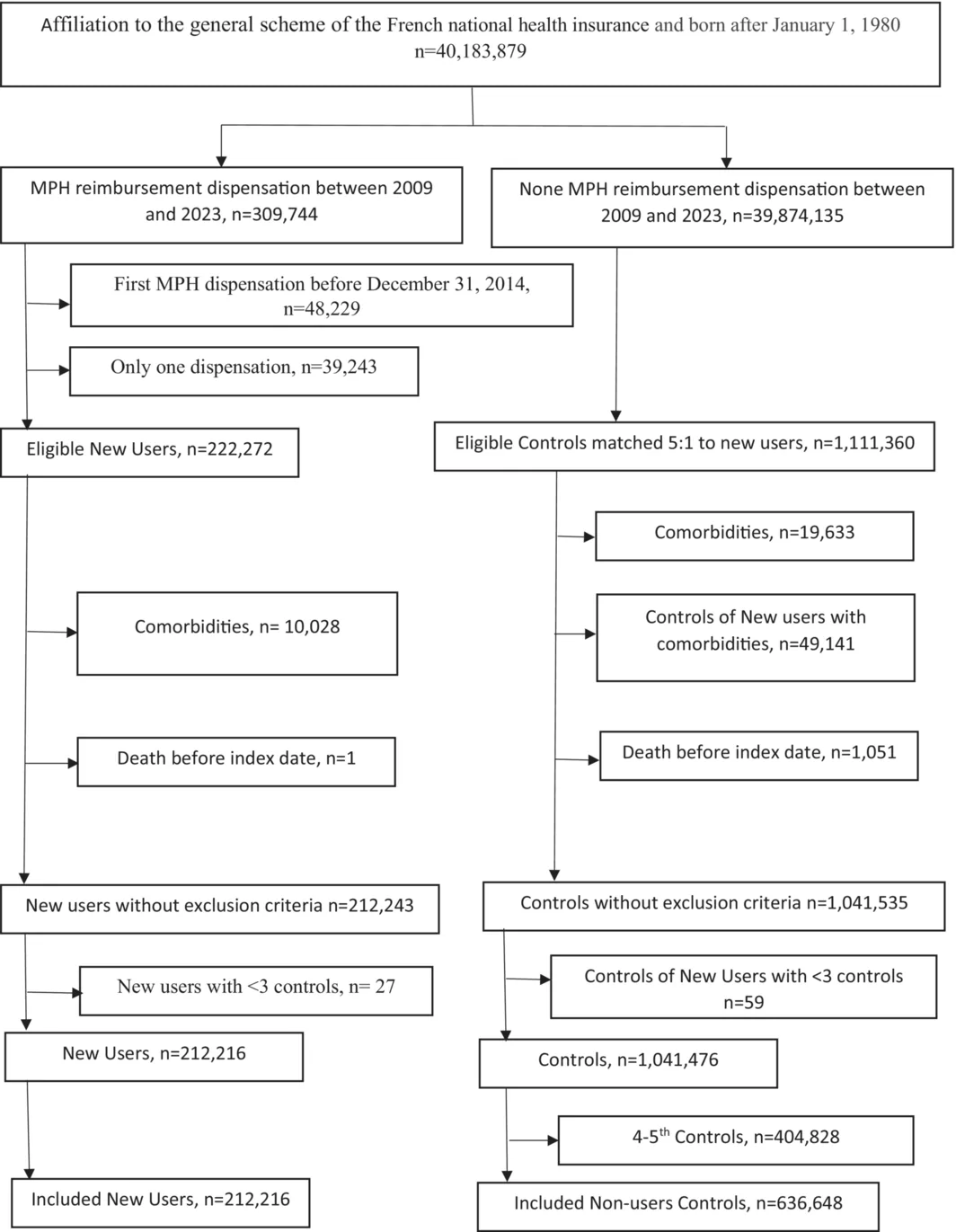
Cohort Flow Chart with sequential exclusion. Sampling included all individuals born between January 1, 1980 and December 31, 2023 in the database. Among them, individuals exposed to methylphenidate (MPH) between 2009 and 2023 were identified. We subsequently excluded individuals with only a single methylphenidate reimbursement dispensation and those with any reimbursement dispensation prior to 2014, in order to restrict the cohort to new users. Due to the large pool of unexposed individuals, five matched unexposed controls (eligible controls) per new user were initially prespecified to enable the exclusion of comorbidities. Matching was performed on sex and date of birth, excluding individuals who had already been selected for a precedent exposed individual. Among eligible individuals, new users and controls with comorbidities were excluded, as well as controls matched to new users with comorbidities. Following this step, three matched unexposed controls (nonusers) were randomly selected for each new user. Accordingly, we excluded new users with fewer than three matched controls, as well as the controls matched to these new users. Next, for new users with more than three matched controls, the fourth and fifth controls were randomly excluded so that only three controls per new user were retained.

### 2.4 Outcome

The study outcome was the occurrence of cardiovascular disease after the index date, identified using ICD‐10 codes for ischemic heart diseases, hypertensive diseases, valve disorders, cardiomyopathy, conduction disorders, cardiac arrest, cardiac arrhythmias, heart failure, cerebrovascular diseases, aneurysm and dissection, and other heart disease (Table S1). Hypertensive diseases were also identified by exposure to antihypertensive drugs (using CIP13 codes). For patients with multiple diagnoses recorded, only the first cardiovascular event was considered to avoid potential bias related to subsequent events being influenced by the first event.

### 2.5 Covariables

The model was adjusted for age, sex, calendar year of index date, diabetes, tobacco use, hyperlipidemia, obesity, antidepressant use, antipsychotic use, QT‐prolonging drug use, and sympathomimetic vasoconstrictors such as nasal decongestants and bronchodilators, in line with previous studies [12, 24, 25]. Tobacco use included patients with relevant ICD‐10 codes related to tobacco use or nicotine replacement therapy (using CIP13 codes). Diabetes, obesity, and hyperlipidemia were identified via ICD‐10 codes and using CIP13 codes for anti‐diabetic drugs and lipid‐lowering drugs (Table S1).

### 2.6 Residual Confounding

To evaluate residual confounding, negative control outcomes were applied [21, 26, 27]. We selected pediatric diagnoses from the 2023 French ICD‐10 classification used for hospital discharge coding. The selection criteria were diagnoses with a nonzero expected incidence in the pediatric population and no a priori expected association with methylphenidate use, such as psychiatric disorders and sleep apnea syndrome. This approach would allow us to quantify the bias arising from differences in healthcare utilization between the two groups and to assess other potential confounding factors inherent to the database. Twenty‐six negative control outcomes were selected to assess the extent of residual confounding and to calibrate the primary estimates: pyelonephritis, pneumonia, meningitis, pneumothorax, in situ neoplasms, diseases of the blood and blood‐forming organs, stomatitis, hernia, psoriasis, Crohn's disease, scoliosis, urolithiasis, torsion of testis, acute appendicitis, peritonitis, paralytic ileus and intestinal obstruction without hernia, diverticulitis, acute pancreatitis, cholecystitis, gastric ulcer with acute with hemorrhage, mastoiditis, hepatitis, acute renal failure, Addisonian crisis, hemolytic‐uremic syndrome and Evans syndrome. Negative control outcomes were identified using ICD‐10 codes (Table S1).

### 2.7 Sample Size Calculation

The reported incidence of cardiovascular events or diseases in children and young adults ranges from 1 to 13 per 100 000 individuals [10, 28, 29]. The observational study with the longest follow‐up (9.5 years) found a hazard ratio (HR) of 1.83 (95% CI [1.10; 3.04]) for the association between methylphenidate exposure and cardiovascular events [12]. Assuming a conservative incidence of 1 event per 100 000 individuals and aiming to detect a risk ratio of 1.83 with *α* = 0.05 and 80% power, the minimum required sample size was estimated to be 201 368 exposed individuals and 604 102 unexposed individuals (1:3 ratio) using a chi‐squared test for comparing proportions.

### 2.8 Statistical Methods

Incidence rates were expressed per person‐year. Baseline characteristics were summarized using frequencies and percentages for categorical variables, and means and standard deviations (SD) for continuous variables, with outliers retained. Cox proportional hazards models estimated the association between methylphenidate exposure and cardiovascular disease, with the proportionality of risk verified using Schoenfeld residuals. No important confounders were excluded from adjustment based on *p*‐value or other variable selection techniques in accordance with methodological guidelines [17] All covariates were included in the adjusted model. Cumulative incidence curves described the primary outcome by exposure status, using the Kaplan–Meier method. Secondary subgroup analyses were performed in adults (≥ 18 years) and children to assess interactions. After Bonferroni correction for one primary and two secondary analyses, statistical significance was set at *p* < 0.017 [30].

To assess residual confounding, negative control analyses were performed using Cox models with identical covariate adjustment. For each analysis, subjects with the outcome of interest prior to the index date were excluded. The resulting log‐HR bias distribution was used for empirical calibration. Empirical confidence interval calibration was performed using the OHDSI package: EmpiricalCalibration package (version 2.1.3) in R [19].

In addition, the E‐value was calculated to assess the potential impact of unmeasured bias using the *EValue* R package. The E‐value represents the minimum strength of association that an unmeasured confounder would need to have with both the exposure and the outcome to fully explain the observed association [31, 32, 33, 34].

Four sensitivity analyses were performed: estimation of a year‐specific HRs, inclusion of individuals with cardiovascular comorbidities, inclusion of those exposed to methylphenidate before 2014 and modification of the index date to the date of the second pharmacy dispensation to account for the immortal time period between the first and second pharmacy dispensations among exposed patients. Statistical analyses were performed using SAS version 9.4 (SAS Institute Inc., Cary, NC, USA) and R version 4.3.3 (R Foundation for Statistical Computing, Vienna, Austria). ChatGPT (OpenAI) was used to assist with coding and to improve the English language and readability of the manuscript.

## 3 Results

### 3.1 Population

A total of 40 183 879 individuals with affiliation to the general scheme of the French national health insurance system and born after January 1, 1980, were identified. Between January 1, 2009, and December 31, 2023, there were 10 154 087 dispensations of methylphenidate. Overall, 309 744 individuals were identified as users and 39 874 135 as nonusers. A total of 48 229 users who had a reimbursed dispensation prior to January 1, 2014, and 39 243 who had only a single reimbursed dispensation were excluded. Among the remaining 222 272 eligible new users, 1 111 360 eligible nonusers were initially matched. After excluding eligible controls with comorbidities, 27 new users could not be matched due to an insufficient number of eligible controls (fewer than 3 nonusers without comorbidities). After applying exclusion criteria and performing matching 1:3, the final cohort included 212 216 methylphenidate new users and 636 648 matched nonusers (Figure 1).

### 3.2 Participants Descriptive Data

At the index date, participants in both groups had a mean age of 13.7 years (SD: 8.2), were predominantly males (72%), children (80.5%), and had a mean follow‐up duration of 4.03 years (SD: 2.92) for new users (range: 1 day to 10 years) and 4.05 years (SD: 2.92) for nonusers (range: 2 days to 10 years; Table 1). Overall follow‐up ranged from a few days to nearly 10 years.

**TABLE 1:** Population description at index date.

	Methylphenidate new users *n* = 212 216	Methylphenidate nonusers *n* = 636 648
Males	153 258 (72%)	459 774 (72%)
Age, years	13.7 ± 8.2	13.7 ± 8.2
Children	170 822 (80.5%)	512 466 (80.5%)
Tobacco use	5198 (2.4%)	6974 (1.1%)
Diabetes	1102 (0.5%)	2738 (0.4%)
Hyperlipidemia	716 (0.3%)	1235 (0.2%)
Obesity	1337 (0.6%)	2488 (0.4%)
Use of antidepressants	29 046 (13.7%)	15 556 (2.4%)
Use of antipsychotics	28 496 (13.4%)	5864 (0.9%)
Use of bronchodilators	90 658 (42.7%)	213 288 (33.5%)
Use of QT‐prolonging drugs	57 536 (27.1%)	141 645 (22.2%)
Use of nasal decongestants	5692 (2.7%)	14 272 (2.2%)
Follow‐up, years	4.0 ± 2.9	4.1 ± 2.9

*Note:* Quantitative variable in years: mean +/− standard deviation. QT‐prolonging drugs increase the QT interval duration on an electrocardiogram, which may raise the risk of arrhythmias.

New users were more frequently users of tobacco, as well as antidepressants, antipsychotics, and bronchodilators than nonusers. The groups were close with respect to obesity, diabetes, hyperlipidemia, nasal decongestant use, and exposure to QT‐prolonging drugs (Table 1).

### 3.3 Outcome Descriptive Data

There were 1960 new users with a cardiovascular disease (9.2 per 1000 individuals), and 1898 nonusers (3.0 per 1000 individuals). The incidence was 2.3 cardiovascular diseases per 1000 person‐years among new users and 0.7 per 1000 person‐years in nonusers. Of the 3858 outcomes observed (1.1/1000 person‐years), the distribution was as follows: hypertensive diseases (0.6/1000 person‐years), cardiac arrhythmias (0.2/1000 person‐years), conduction disorders (0.1/1000 person‐years), cerebrovascular diseases (0.1/1000 person‐years), heart failure (0.05/1000 person‐years), valve disorders (0.04/1000 person‐years), ischemic heart diseases (0.03/1000 person‐years), cardiac arrest (0.03/1000 person‐years), aneurysm and dissection (0.03/1000 person‐years), cardiomyopathy (0.02/1000 person‐years), and other heart disease (0.02/1000 person‐years).

#### 3.3.1 Primary Outcome Analysis

The estimated adjusted HR of cardiovascular diseases was 2.37 (95% CI [2.21; 2.54]), *p*‐value < 0.017 (Table 2). The confounding factor with the highest adjusted HR was antipsychotics use before the index date. The cumulative incidence curves indicate a higher cumulative incidence of cardiovascular diseases over time in new users compared to nonusers (Figure 2). The Schoenfeld residuals plots were horizontal, confirming the proportional hazards assumption (Figure S1).

**TABLE 2:** Cox model‐estimated hazard ratios for primary outcome.

	No. of CV diseases (*n* = 3858)	Follow‐up,^a^ no. of person‐years (*n* = 3 435 986)	Crude CV diseases incidence rate per 1000 person‐years	CV diseases unadjusted estimate HR (95% CI)	CV diseases adjusted^b^ estimate HR (95% CI)
Methylphenidate new users	1960	855 362	2.29	3.12 (2.92; 3.32)	2.37 (2.21; 2.54)
Females	1115	833 378	1.34	1.26 (1.18; 1.36)	0.93 (0.86; 1.00)
Age	***	***	***	1.07 (1.06; 1.07)	1.06 (1.05; 1.06)
Calendar year of index date	***	***	***	0.62 (0.54; 0.72)	1.19 (1.02; 1.39)
Hyperlipidemia	49	5237	9.36	8.36 (6.31; 11.08)	1.90 (1.42; 2.55)
Tobacco use	200	25 553	7.83	7.23 (6.27; 8.35)	1.39 (1.19; 1.63)
Diabetes	53	10 382	5.11	4.56 (3.48; 5.98)	1.70 (1.28; 2.26)
Obesity	73	10 546	6.92	6.21 (4.93; 7.83)	2.11 (1.65; 2.69)
Use of Antidepressants	648	112 899	5.74	5.95 (5.47; 6.48)	1.31 (1.17; 1.47)
Use of bronchodilators	1329	1 001 911	1.33	1.28 (1.20; 1.37)	1.21 (1.13; 1.30)
Use of nasal decongestants	153	48 891	3.13	2.84 (2.41; 3.34)	0.97 (0.82; 1.15)
Use of antipsychotics	681	118 587	5.74	5.99 (5.51; 6.51)	2.30 (2.08; 2.54)
Use of QT‐prolonging drugs	1115	734 549	1.52	1.51 (1.41; 1.62)	1.37 (1.27; 1.47)

Abbreviations: CI, confidence interval; CV, cardiovascular; HR, hazard ratio; No, number.

^a^ Follow‐up of the entire specific subgroup in person‐years.

^b^ Adjusted for sex, age, calendar year of index date, hyperlipidemia, tobacco use, diabetes, obesity, and use of antidepressants, bronchodilators, nasal decongestants, antipsychotics, and QT‐prolonging drugs.

*** Not applicable for a continuous variable.

**FIGURE 2:**
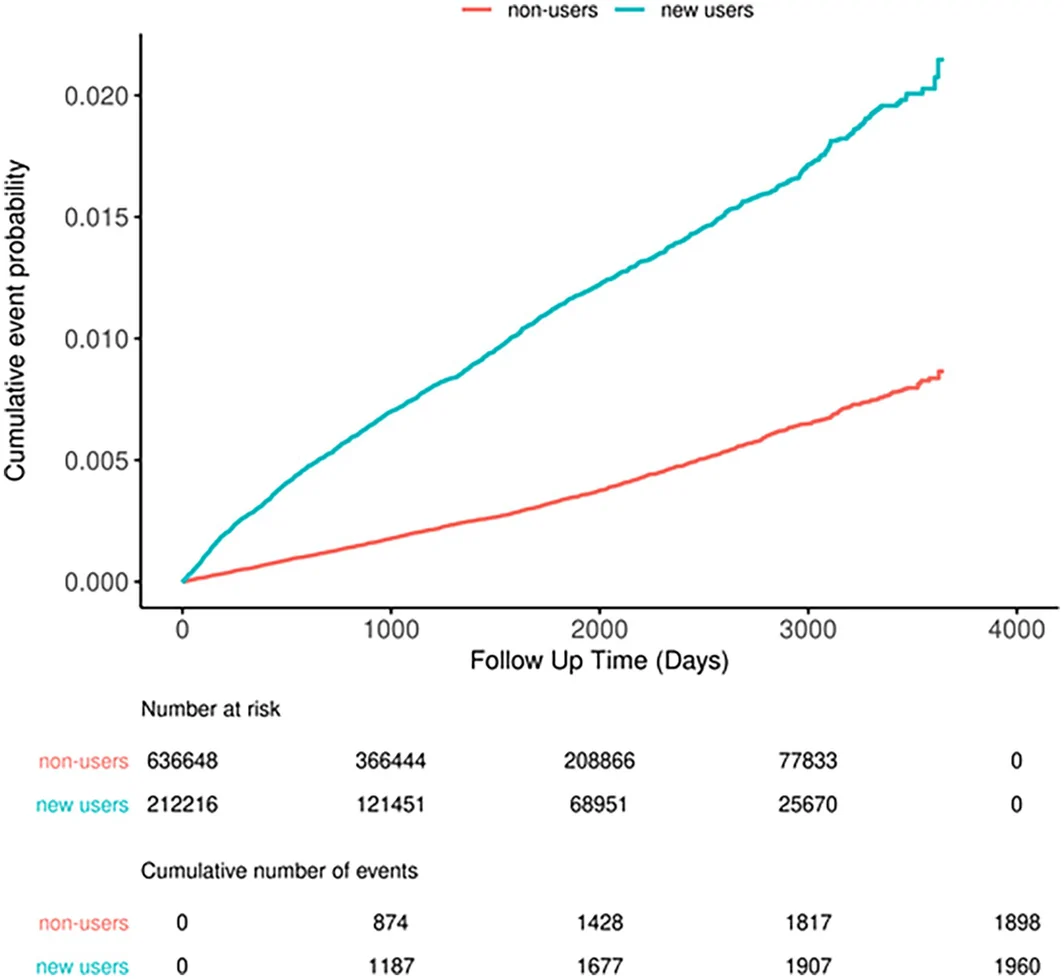
Adjusted cumulative incidence curves for the time to diagnosis of cardiovascular diseases. Cumulative event probability, follow up time (days).

#### 3.3.2 Negative Control Outcomes and Residual Bias

Among the 26 negative control outcomes assessed, methylphenidate exposure was associated with a significantly increased risk in 13, and no statistically significant association in 13; none was associated with a significant reduction in risk (Figure 3). This findings indicate the presence of residual bias.

**FIGURE 3:**
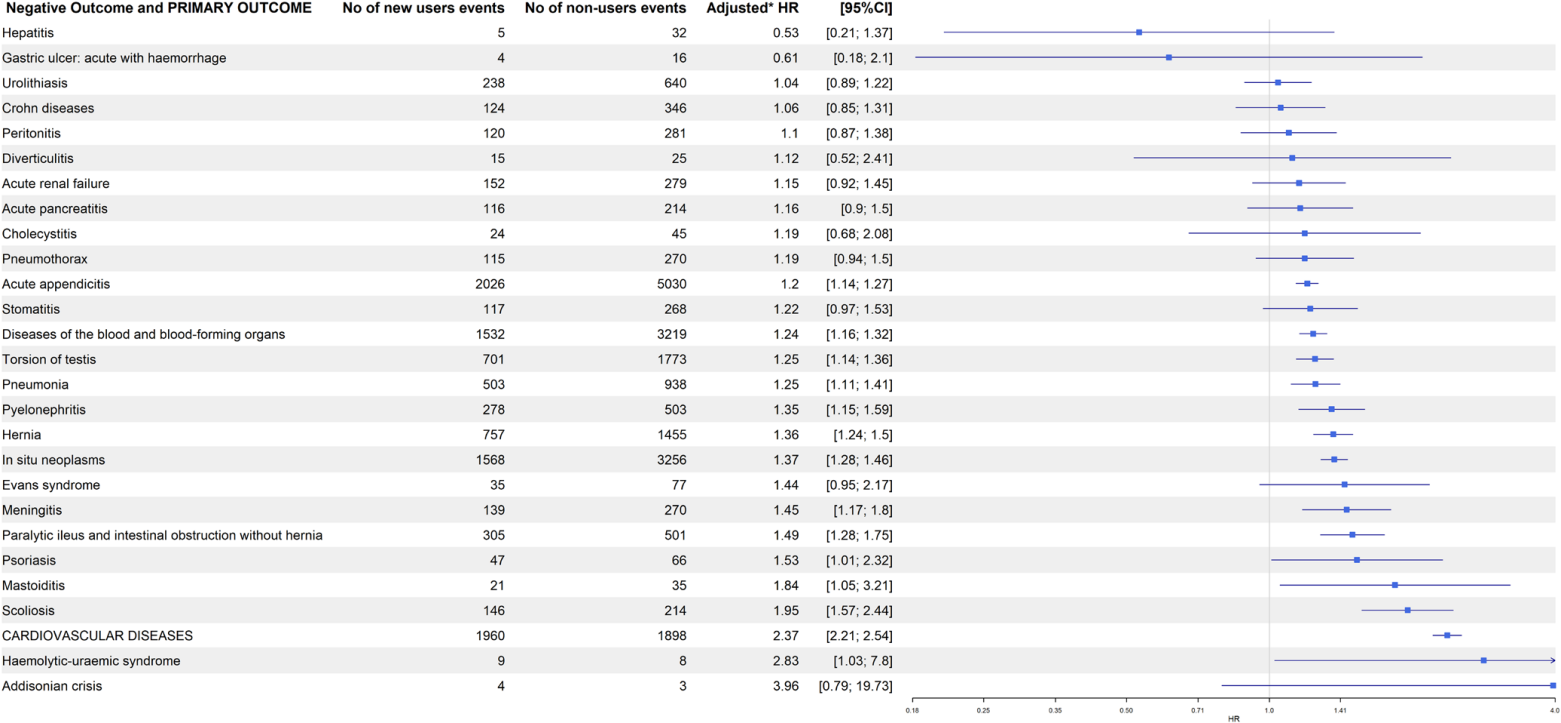
Forest plot of cox model‐estimated hazard ratios for negative control outcome. *Adjusted for sex, age, calendar year of index date, hypercholesterolemia, tobacco use, diabetes, obesity, and use of antidepressants, bronchodilators, nasal decongestants, antipsychotics, and QT‐prolonging drugs.

The overall distribution of these confidence intervals across all negative control outcomes was then used to derive a calibrated confidence interval, yielding a hazard ratio of cardiovascular diseases in the methylphenidate‐exposed group of 1.85 (95% CI [1.55; 2.22]). The *E*‐value for the observed hazard ratio (adjusted HR = 2.37) was 4.17. The *E*‐value for the lower bound of the 95% confidence interval (2.21) was 3.84.

### 3.4 Secondary Analysis

Subgroup analyses showed an adjusted HR of cardiovascular diseases of 2.75 (95% CI [2.54; 2.98]) among 683 288 children (*p*‐value < 0.017) and 1.56 (95% CI [1.38; 1.77]) among 165 576 adults (*p*‐value < 0.017) in the methylphenidate‐exposed group. Children accounted for 80.5% of the database, while adults represented 19.5%.

### 3.5 Sensitivity Analyses

No meaningful variation was observed in the HRs of cardiovascular diseases in the methylphenidate new user group across index calendar years (Table S2). Including patients previously excluded due to pre‐2014 exposure resulted in an adjusted HR of 2.46 (95% CI [2.33; 2.59]). Similarly, retaining individuals with cardiovascular comorbidities yielded a HR of 2.47 (95% CI [2.32; 2.63]). When the index date in the primary analysis was redefined as the date of the second pharmacy dispensing, the estimated effect was adjusted HR 2.49 (95% CI [2.30; 2.68]).

## 4 Discussion

In this large nationwide cohort study, methylphenidate use was associated with a significantly increased risk of cardiovascular disease compared with nonusers. This association remained consistent across sensitivity and subgroup analyses; notably, despite variations in follow‐up duration across calendar year‐specific analyses, HR remained consistent. The observed cardiovascular diseases were rare overall, with incidence rates of 2.3 per 1000 person‐years in the new users group versus 0.7 in the control group. This finding illustrates that, although an increased risk was observed, its magnitude remains very small, primarily because the baseline risk is extremely low.

Our findings agree with the other long‐term study, identifying the same two most frequent conditions in the overall sample. Hypertensive disorders were the most frequent outcomes (0.6/1000 person‐years), followed by arrhythmias (0.2/1000 person‐years), consistent with the other study, in which hypertension (8%) and arrhythmias (23%) were also the most common [12].

The present study contributes to the growing body of evidence on the long‐term cardiovascular risk of methylphenidate, helping to reconcile previously contradictory findings. For example, the Danish cohort study, with a mean follow‐up of 9.5 years, found an 83% increased risk of cardiovascular events associated with methylphenidate use [12]; the Swedish cohort study, with a median follow‐up of 4.1 years (interquartile range: 1.9–6.8 years), found a 27% (95% CI [17%; 39%]) increased risk of cardiovascular events among individuals exposed to methylphenidate for 3 to 5 years, and a 23% (95% CI [12%; 36%]) increased risk for those with exposure exceeding 5 years [11]. However, the Taiwanese cohort study, with 7.5 years of follow‐up, did not find an increased risk of major cardiovascular events among patients treated with methylphenidate for more than 7 days compared to non‐ADHD medication users (HR 0.85; 95% CI [0.72–0.99]) [16]. Taken together these studies alone are insufficient to draw conclusions. They did not assess residual bias, which was done herein providing more robust estimates in the presence of potential unmeasured or residual confounding. After calibration of the confidence intervals, the association remained significant, supporting the robustness of this finding. Another strength of the study was the inclusion of incident users. Furthermore, variables were measured consistently across both study groups, and those with a prior cardiovascular disease before the index date were excluded to measure more accurately the incidence of these; this was also applied to the analysis of negative control outcomes.

Our result is supported by a physiological rationale. Methylphenidate is a central nervous system stimulant structurally related to amphetamines through its classification as a phenethylamine derivative. Nevertheless, it is distinguished by its specific chemical scaffold, a benzylpiperidine, and by its pharmacological mechanism of action, which is primarily the inhibition of dopamine and norepinephrine reuptake rather than the promotion of their release. Amphetamines are known to increase blood pressure and heart rate. Studies have shown that methylphenidate increases heart rate and blood pressure in the short term [24, 35]. Sustained elevations in these parameters may contribute to the development of cardiovascular disease [31, 36, 37, 38].

Owing to its observational nature, the present study is inherently susceptible to bias and the potential for spurious associations. Moreover, data only reflect methylphenidate reimbursement dispensation rather than confirmed intake, increasing the risk of exposure misclassification and potentially diluting true treatment effect. To reduce the risk of exposure misclassification, only individuals who had received two dispensations of methylphenidate were included. We attempted to measure exposure, outcome and covariates as accurately as possible. Among covariates that should have been adjusted for, data on family history and exposure to illicit drugs were not available. Regarding outcome measurement, individuals with hypertension who had neither a hospitalization with an ICD‐10 code for hypertensive diseases nor reimbursed antihypertensive treatment were not captured. It is unclear whether this outcome misclassification was similar between the two groups; however, if events were more frequent in the new‐user group, this misclassification would likely bias the effect estimate toward the null. This potential information bias would not be expected to increase the risk of a false‐positive association, in contrast to confounding by indication. If ADHD increases cardiovascular risk, there would be confounding by indication in our study because our matched controls were not selected based on ADHD status. We hypothesized that there was no association between cardiovascular risk and ADHD; however, to the best of our knowledge, this has not been investigated outside of ADHD medications. The observed association between treatment exposure and negative control outcomes suggests the presence of residual confounding. Residual bias may partly reflect differences in healthcare utilization between groups. More frequent healthcare visits among methylphenidate users may increase the detection of all conditions, particularly those with subtle or minimal symptoms, such as scoliosis, independently of any treatment effect. In our study, weighting the results to account for the effect attributable to residual bias allows this source of bias to be taken into consideration. In future studies, controls could be restricted to healthcare users with at least two medical visits per year or another medication use during follow‐up to reduce bias arising from differential healthcare utilization between groups. Another potential information bias stems from the limited follow‐up duration, which may not have allowed all events to occur. Given that the mean follow‐up was 4 years, the true long term effect may be larger over an extended time horizon.

## 5 Conclusion

This study suggests a possible long‐term association between methylphenidate use and cardiovascular diseases. These findings support the need for continued clinical vigilance, in line with existing recommendations for routine monitoring of blood pressure and heart rate in patients receiving methylphenidate. Further research is needed to better characterize this potential risk, particularly through studies incorporating dose–response analyses, more detailed classification of cardiovascular outcomes, and longer follow‐up periods.

## Author Contributions

Study concept and design: Christelle Berthod, Thomas Loppinet, Behrouz Kassaï‐Koupai, and Matthias Jacquet‐Lagreze. Acquisition of data: Christelle Berthod, Thomas Loppinet, Marietou Ndiaye, and Inesse Boussaha. Analysis/interpretation of data: Christelle Berthod, Thomas Loppinet, Behrouz Kassaï‐Koupai, and Matthias Jacquet‐Lagreze. Drafting of the manuscript: Christelle Berthod. Critical revision of the manuscript: Christelle Berthod, Thomas Loppinet, Behrouz Kassaï‐Koupai, and Matthias Jacquet‐Lagreze. Statistical analysis: Thomas Loppinet.

## Funding

The authors have nothing to report.

## Ethics Statement

The study protocol was approved by *Institut National de la Santé et de la Recherche Médicale Française* (INSERM). There is very minimal risk as no intervention is involved in the study design. Subjects' privacy and confidentiality were assured; no identifiers were collected. The data management complied with French law on personal data protection (*Article R. 1461‐12 du Code de la santé publique*).

## Consent

In accordance with French regulations, informed consent was not required for register‐based studies. The need for written informed consent was waived by the INSERM IRB. To ensure privacy and confidentiality, no identifiable information was collected. Data security measures included encryption of patient identifiers and restricted access to the dataset, adhering to ethical guidelines and standards.

## Conflicts of Interest

The authors declare no conflicts of interest.

## Supporting information


**Table S1:** Coding of variables using CIP13 and ICD‐10 codes.
**Table S2:** Hazard ratios stratified by year of inclusion.
**Figure S1:** Schoenfeld residues of Cox model‐estimated hazard ratios for primary outcome.

## Data Availability

The SNDS database is not publicly available because it contains sensitive patient‐level information. Access to the data is subject to strict regulatory requirements and must be granted in accordance with French law through the Health Data Hub (https://www.health‐data‐hub.fr/).
