# Supplementary material for: Methylphenidate and Long‐Term Risk of Cardiovascular Diseases: A Nationwide Cohort Study
Source: Pharmacoepidemiol Drug Saf. 2026 Oct 11;35(10):e70493. doi: 10.1002/pds.70493 (PMC13655642; doi:10.1002/pds.70493)
Supplement: Supplementary file 1 [file PDS-35-e70493-s001.docx]

Table S1: Coding of variables using CIP13 and ICD-10 codes

| Exposition variable | Measure Methods |
| --- | --- |
| Methylphenidate | **CIP13 in DCIR:** FROM ORAVUE.ER_PHA_F WHERE pha.PHA_PRS_C13 IN (**3400936155411**,**3400938732207**,**3400957322458**,**3400936155589**,**3400936155640**,**3400949478026**,**3400949478255**,  **3400949481385**,**3400949481446**,**3400949478316**,**3400949478484**,**3400949481507**,**3400949481675**,**3400949481736**,  **3400949481965**,**3400949482108**,**3400949482276**,**3400949477883**,**3400949477944**,**3400949478545**,**3400949478606**,  **3400930227886**,**3400955081258**,**3400930227893**,**3400955081265**,**3400930227909**,**3400955081272**,**3400930227916**,  **3400955081296**,**3400930270998**,**3400930270448**,**3400930271018**,**3400930270455**,**3400930271025**,**3400930270462**,  **3400930264171**,**3400930264188**,**3400930264201**,**3400936371750**,**3400936371811**,**3400936371989**,**3400936372061**,  **3400936372122**,**3400936372290**,**3400936372351**,**3400936372412**,**3400936372580**,**3400936372641**,**3400936371170**,  **3400936371231**,**3400936371460**,**3400936371521**,**3400936371699**,**3400930108734**,**3400955038856**,**3400955038863**,  **3400958684470**,**3400955038870**,**3400930108741**,**3400955038887**,**3400958684531**,**3400955038894**,**3400930108758**,  **3400958684760**,**3400955038900**,**3400930188491**,**3400930188507**,**3400930188521**,**3400928001306**,**3400928001474**,  **3400928001535**,**3400928001764**,**3400928001825**,**3400928001993**,**3400958514708**,**3400958514937**,**3400958515187**,  **3400937761789**,**3400949726165**,**3400937761840**,**3400957026813**,**3400957026981**,**3400937761901**,**3400949726226**,  **3400937762090**,**3400937831321**,**3400957027124**,**3400937762151**,**3400949726745**,**3400937762212**,**3400937831499**,  **3400933942410**,**3400933942649**,**3400933929404**,**3400941686535**,**3400941686764**,**3400957974572**,**3400936534933**,  **3400936231382**,**3400956496914**,**3400936535015**,**3400936231504**,**3400956497164**,**3400936535183**,**3400936231733**,  **3400956497225)** |
| Covariables | **Measure Methods** |
| Sex | Males (1), Females (2) |
| Age | Date index - (15, month of birth, year of birth) |
| Tobacco use | **ICD-10:** Z5731, Z772, Z716, Z720, Z87891, Z1389, P9681, T652, F17  **CIP13 in DCIR:**from ORAVUE.IR_PHA_R  where PHA_EPH_LIB = "MEDICAMENTS POUR L'ARRET DU TABAC" |
| Diabetes | ICD-10: E14  CIP13 in DCIR:from ORAVUE.IR_PHA_R  where PHA_ATC_L03 in ("MEDICAMENTS DU DIABETE" |
| Hyperlipidemia | ICD-10: E780  CIP13 in DCIR:from ORAVUE.IR_PHA_R  where PHA_ATC_L03 in ("AGENTS MODIFIANT LES LIPIDES") |
| Obesity | ICD-10: E660 |
| Use of Antidepressants | CIP13 in DCIR:from ORAVUE.IR_PHA_R  where PHA_EPH_LIB in (  "ANTIDEPRESSEURS INHIBITEURS DE RECAPTURE DE SEROTONINE ET NORADRENALINE",  "ANTIDEPRESSEURS INHIBITEURS SELECTIFS DE LA RECAPTURE DE SEROTONINE (SSRI)","AUTRES ANTIDEPRESSEURS" |
| Use of Antipsychotics | CIP13 in DCIR:from ORAVUE.IR_PHA_R  where PHA_EPH_LIB in (  "ANTIPSYCHOTIQUES ATYPIQUES",  "ANTIPSYCHOTIQUES CONVENTIONNELS" |
| Use of Bronchodilators | CIP13 in DCIR:from ORAVUE.IR_PHA_R  where PHA_ATC_L03 in ("MEDICAMENTS POUR LES SYNDROMES OBSTRUCTIFS DES VOIES AERIENNES") |
| Use of QT prolonging drugs | CIP13 in DCIR:from ORAVUE.IR_PHA_R t1  WHERE t1.PHA_ATC_LIB in ("AMIODARONE", "DISOPYRAMIDE", "DRONEDARONE", "FLECAINIDE", "HYDROQUINIDINE", "QUINIDINE", "SOTALOL", "OXIPLATINE", "VANDETANIB", "DOMPERIDONE",  "DROPERIDOL", "ONDANSETRON", "CHLOROQUINE", "HYDROXYCHLOROQUINE", CHLORPROMAZINE", "HALOPERIDOL", "LEVOMEPROMAZINE", "PIMOZIDE", "SULPIRIDE", "DONEPEZIL", "METHADONE", "ANAGRELIDE", "PENTAMIDINE", "TERLIPRESSINE", "PAPAVERINE" |
| Use of Nasal decongestants | CIP13 in DCIR: from ORAVUE.IR_PHA_R  where PHA_EPH_LIB  in("DECONGESTIONNANTS A USAGE NASAL" |
| Follow-up | Mean of date of outcome or censored at the date of death or end of our observation (December 31, 2023) – Index date |

| Exclusion Criteria | Measure Methods with ICD-10 |
| --- | --- |
| Sleep apnoea | G47.3 |
| Congenital malformations of the circulatory system | Q20-Q28 |
| Down syndrome | Q90 |
| Fragile X | Q99.2 |
| Glomerular diseases | N00-N08 |
| Kawasaki disease | M30.3 |
| Paediatric Inflammatory Multisystem Syndrome | U10.9 |
| Outcome: Cardiovascular diseases | **Measure Methods with ICD-10 and CIP13** |
| Ischaemic heart diseases | I20-21,I24-25 |
| Hypertensive diseases | I10-I15  antihypertensive drugs (code CIP13):  from ORAVUE.IR_PHA_R  where PHA_ATC_L03 in ("ANTIHYPERTENSEURS") |
| Valve disorders | I34-I37 |
| Cardiomyopathy | I42 |
| Conduction disorders | I44-I45 |
| Cardiac arrest | I46 |
| Cardiac arrhythmias | I47-I49 |
| Heart failure | I50 |
| Cerebrovascular diseases | I60-I69 |
| Other heart disease | I51-I52 |
| Negative control Outcome | **Measure Methods with ICD-10** |
| Pyelonephritis | N10,N136 |
| Pneumonia | J13, J14, J15, J16, J17, J18 |
| Meningitis | A87, A390, G00, G01, G02, G03 |
| Pneumothorax | J93 |
| In situ neoplasms | D0, D1, D2, D3, D4 |
| Diseases of the blood and blood-forming organs | D5, D6, D7, D8 |
| Stomatitis | K12 |
| Hernia | K41, K42, K43, K44, K45, K46 |
| Psoriasis | L40 |
| Scoliosis | M41 |
| Urolithiasis | N20, N23 |
| Torsion of testis | N44 |
| Acute appendicitis | K35 |
| Peritonitis | K65 |
| Paralytic ileus and intestinal obstruction without hernia | K56 |
| Diverticulitis | K572, K575 |
| Acute pancreatitis | K85 |
| Cholecystitis | K810 |
| Gastric ulcer: acute with haemorrhage | K250 |
| Mastoiditis | H700 |
| Hepatitis | B15 |
| Acute renal failure | N17 |
| Addisonian crisis | E272 |
| Haemolytic-uraemic syndrome | D593 |
| Evans syndrome | D693 |
| Crohn disease | K50 |

CIP13: : the 13-digit drug identifier code (*Code Identifiant de Présentation)*

DCIR: Inter Regime Consumption Data (*Données de Consommation InterRégime*)

In Inter Regime Consumption Data (IRCD) in the table IR_PHA_R, we use variables PHA_EPH_LIB, PHA_ATC_LIB, PHA_ATC_L03 to identification medication.

**Table S2. Hazard Ratios Stratified by Year of Inclusion**

| Index date year | Crude CV diseases  New Users incidence rate per 1000 Person-years | | | Crude CV diseases  No users incidence rate per 1000  Person-years | | Adjusted* HR | | [95%CI] |
| --- | --- | --- | --- | --- | --- | --- | --- | --- |
| 2014 | | 1.89 | 0.80 | | 1.94 | | [1.64; 2.28] | |
| 2015 | | 1.94 | 0.77 | | 2.00 | | [1.68; 2.37] | |
| 2016 | | 2.17 | 0.72 | | 2.37 | | [1.98; 2.83] | |
| 2017 | | 2.28 | 0.67 | | 2.58 | | [2.14; 3.12] | |
| 2018 | | 2.42 | 0.67 | | 2.75 | | [2.25; 3.35] | |
| 2019 | | 2.43 | 0.62 | | 3.01 | | [2.44; 3.73] | |
| 2020 | | 2.28 | 0.67 | | 2.52 | | [2.00; 3.18] | |
| 2021 | | 2.39 | 0.82 | | 2.10 | | [1.68; 2.63] | |
| 2022 | | 3.01 | 0.89 | | 2.37 | | [1.87; 3.01] | |
| 2023 | | 4.74 | 0.91 | | 3.52 | | [2.52; 4.93] | |

CV: Cardiovascular

*Adjusted for sex, age, calendar year of index date, hypercholesterolemia, tobacco use, diabetes, obesity and use of antidepressants, bronchodilators, nasal decongestants, antipsychotics and QT-prolonging drugs.

**Figure S1: Schoenfeld residues of** **Cox Model-Estimated Hazard Ratios for Primary Outcome**
